# Invasive Pneumococcal Disease in Healthy Adults: Increase of Empyema Associated with the Clonal-Type Sweden^1^-ST306

**DOI:** 10.1371/journal.pone.0042595

**Published:** 2012-08-13

**Authors:** Imma Grau, Carmen Ardanuy, Laura Calatayud, Dora Rolo, Arnau Domenech, Josefina Liñares, Roman Pallares

**Affiliations:** 1 Infectious Disease Department, Hospital Bellvitge, Idibell, Ciberes, University of Barcelona, Barcelona, Spain; 2 Microbiology Department, Hospital Bellvitge, Idibell, Ciberes, University of Barcelona, Barcelona, Spain; Health Protection Agency, United Kingdom

## Abstract

**Background:**

Adult invasive pneumococcal disease (IPD) occurs mainly in the elderly and patients with co-morbidities. Little is known about the clinical characteristics, serotypes and genotypes causing IPD in healthy adults.

**Methods:**

We studied 745 culture-proven cases of IPD in adult patients aged 18–64 years (1996–2010). Patients were included in two groups: 1.) adults with co-morbidities, and 2.) healthy adults, who had no prior or coincident diagnosis of a chronic or immunosuppressive underlying disease. Microbiological studies included pneumococcal serotyping and genotyping.

**Results:**

Of 745 IPD episodes, 525 (70%) occurred in patients with co-morbidities and 220 (30%) in healthy adults. The healthy adults with IPD were often smokers (56%) or alcohol abusers (18%). As compared to patients with co-morbidities, the healthy adults had (P<0.05): younger age (43.5+/−13.1 vs. 48.7+/−11.3 years); higher proportions of women (45% vs. 24%), pneumonia with empyema (15% vs. 7%) and infection with non-PCV7 serotypes including serotypes 1 (25% vs. 5%), 7F (13% vs. 4%), and 5 (7% vs. 2%); and lower mortality (5% vs. 20%). Empyema was more frequently caused by serotype 1. No death occurred among 79 patients with serotype 1 IPD. There was an emergence of virulent clonal-types Sweden^1^-ST306 and Netherlands^7F^-ST191. The vaccine serotype coverage with the PCV13 was higher in healthy adults than in patients with co-morbidities: 82% and 56%, respectively, P<0.001.

**Conclusion:**

In this clinical study, one-third of adults with IPD had no underlying chronic or immunosuppressive diseases (healthy adults). They were often smokers and alcohol abusers, and frequently presents with pneumonia and empyema caused by virulent clones of non-PCV7 serotypes such as the Sweden^1^-ST306. Thus, implementing tobacco and alcohol abuse-cessation measures and a proper pneumococcal vaccination, such as PCV13 policy, in active smokers and alcohol abusers may diminish the burden of IPD in adults.

## Introduction

*Streptococcus pneumoniae* remains a major cause of morbidity and mortality worldwide. The pneumococcus, with more than 93 serotypes, usually colonizes the nasopharynx of young children, and more infrequently of adults, and occasionally can invade the lungs, bloodstream or brain causing severe infections [1]–[7].

Invasive pneumococcal disease (IPD) occurs mainly in young children and older adults (≥65years) as well as in patients with co-morbidities (e.g., chronic lung disease, cardiovascular disease, cirrhosis, malignancies, HIV, asplenia) [5], [6]. Although several case reports exist, little is known about the characteristics of IPD in healthy adults [6]. Some reports have also shown that young people who are smokers or alcohol abusers may be at higher risk of IPD [5], [8].

The prevention of adult IPD has been based on the pneumococcal polysaccharide vaccine 23-valent (PPV23), which is recommended for the elderly and patients with co-morbidities [9]. However, its immunologic response and effectiveness may not be optimal in some populations, particularly in immunocompromised patients [10]. The 7-valent pneumococcal conjugate vaccine (PCV7), which usually produces a better immunologic response and may reduce acquisition of nasopharyngeal carriage, was introduced in the year 2000 for children in the USA [11] and it was followed by a decline in IPD caused by vaccine serotypes in children as well as in adults - through an indirect effect (herd protection) - [11], [12]. The recent introduction of the PCV10 and PCV13 for children and currently for adults may change significantly the IPD epidemiology. However, the consequences of the “replacement phenomenon”, -that means the emergence of serotypes not contained in the conjugate vaccines- [13], [14], are unknown.

Thus, it is important to know the serotypes causing IPD, including in healthy adults, in order to look for new target populations for implementing future vaccination strategies.

The objectives of our study were to analyze the clinical presentation, serotypes and clonal-type distribution, and mortality of IPD in healthy adults (aged 18–64 years).

## Methods

We have collected data on all patients with IPD at our institution (Hospital Bellvitge, University of Barcelona) over the past three decades. A detailed description of the methodology was reported elsewhere [15]–[17].

For the present report, we studied all IPD episodes seen during the last 15 years (1/1996–12/2010) in adults aged 18–64 years. Patients were included in two groups: “healthy adults” and “patients with co-morbidities”.

### Definitions

Culture-proven IPD was defined according to the clinical findings and the isolation of *Streptococcus pneumoniae* from a normally sterile body fluid such as blood, pleural fluid, cerebrospinal fluid (CSF), peritoneal fluid or joint fluid. Pneumococcal pneumonia was diagnosed in patients with signs or symptoms of an acute lower respiratory tract infection together with a new pulmonary infiltrate on chest radiograph. For the diagnosis of empyema, isolation of *S.pneumoniae* from the pleural fluid was required. Other origins of IPD (e.g., meningitis, peritonitis, arthritis) were diagnosed based on standard criteria.

Alcohol abuse was considered when the patient reported a daily alcohol intake of 80 gr. or 60 gr. for men and women, respectively, at least during the previous year. Current smoking was considered when the patient has been smoking at least ten cigarettes per day during the last year. It should be in mind that self reporting of alcohol and smoking habits is notoriously unreliable, and tend to underestimate consumption.

Special care was taken in assessing smokers and alcohol abusers to rule out associated co-morbidities. Prior antibiotic therapy was defined as the intake of any antibiotic for more than 48 hours during the previous 3 months.

We considered patients to be “healthy adults” when they had no prior or coincident diagnosis of a chronic or immunosuppressive underlying disease. Otherwise, “patients with co-morbidities” were those who had a confirmed diagnosis of one or more of the following: chronic pulmonary diseases (e.g., chronic obstructive pulmonary disease [COPD], emphysema, bronchiectasis, asthma, interstitial lung disease); cardiovascular diseases, chronic liver disease, diabetes, malignant disease, HIV infection, chronic rheumatic diseases or vasculitis, end stage chronic renal failure, cerebrovascular or degenerative brain diseases, asplenia, transplant recipients (bone marrow and solid-organ transplants), and long-term use of corticosteroids or other immunosuppressive therapies.

The diagnosis of septic shock was based on a systolic blood pressure below 90 mm Hg and peripheral hypoperfusion together with clinical and/or bacteriological evidence of uncontrolled infection. Antibiotic therapy was prescribed according to the hospital guidelines being the most frequent schedules for pneumonia: ceftriaxone, levofloxacin or macrolides, alone or in combination; and for meningitis: cefotaxime with or without vancomycin. Mortality was recorded when the patient died within 30 days of diagnosis.

Ethical statement. This study and publication of the results were approved by the “Institutional Review Board - Comité Ètic d'Investigació Clínica del Hospital Universitari de Bellvitge”, and the Institutional Review Board specifically waived the need for consent because the study was retrospective and the source of data was anonymized.

### Microbiological methods

*Streptococcus pneumoniae* strains were identified by conventional methods (optochin susceptibility and bile solubility). Pneumococci were serotyped by dot-blot assay or Quellung reaction at the Spanish Reference Laboratory, Majadahonda, Madrid, Spain.

We evaluated the vaccine serotype coverage of **PCV7** (serotypes 4, 6B, 9V, 14, 18C, 19F, 23F), **PCV10** (additional 1, 5, 7F) and **PCV13** (additional 3, 6A, 19A). In Spain, pneumococcal conjugate vaccines for children were introduced as follows: PCV7 in June 2001, PCV10 in May 2009 and PCV13 in June 2010.

Antimicrobial susceptibility to penicillin, cefotaxime, ceftriaxone, erythromycin, tetracycline, ciprofloxacin and co-trimoxazole were tested by microdilution method (Sensititre) following the Clinical Laboratory Standard Institute (CLSI) methods [18]. *S. pneumoniae* ATCC 6303 and *S. pneumoniae* ATCC 49619 were used as control strains. Antimicrobial resistances were determined according to the CLSI criteria [19].

Pulsed-field-gel-electrophoresis (PFGE) was performed to all available isolates. Genomic DNA embedded in agarose plugs was restricted with *Sma*I (New England BioLabs) and fragments were separated by PFGE in a CHEF-DRIII apparatus (Bio-Rad). Multilocus sequence typing (MLST) was carried out as described previously [20] on selected strains that were representative isolates of PFGE clusters shared by three or more isolates. The allelic number and sequence types (ST) were assigned using the pneumococcal MLST web site http://spneumoniae.mlst.net/, which is located at Imperial College London and is funded by the Wellcome Trust. When an unusual association between serotype and sequence type was found, the serotype was confirmed by PCR, using previously described methodology (http://www.cdc.gov/ncidod/biotech/strep/pcr.htm), and MLST was repeated.

### Statistical analysis

Statistical analysis was carried out with the PASW-18. We used Chi-square test or Fisher's exact test for categorical variables (two-by-k contingency tables) and Student t test for continuous variables. Analyses were adjusted by means of multiple logistic regression models in order to determine those variables independently associated with healthy adults (compared to patients with co-morbidities). All *P* values were two-tailed and P<0.05 were considered statistically significant. To calculate the incidence rates we used as a denominator the number of persons, by age group per year, in the public database of the Web “Official Statistics in Catalonia” [21].

## Results

Between 1/1996 and 12/2010, we studied 1414 IPD episodes in adult patients, in whom *S.pneumoniae* was isolated from one or more sterile site: blood 1320, pleural fluid 128, CSF 107, abdominal fluid 68, and joint fluid 2.

Out of 1414 IPD episodes, 745 (53%) occurred in adults (aged 18–64 years) and 669 (47%) in elderly (≥65 years). Out of 745 IPD episodes in adults, 525 (70%) occurred in patients with co-morbidities and 220 (30%) in healthy adults. The co-morbidities diagnosed in our patients are shown at the foot of Table 1.

**Table 1:** Characteristics of 745 invasive pneumococcal disease episodes in adults (aged 18–64 years): A comparison between patients with co-morbidities and healthy adults.

	Healthy N = 220	Co-morbidities* N = 525	*P* Value
Age, mean+/−SD, years	43.53 (+/−13.1)	48.78 (+/−11.3)	<0.001
18–49 yrs	134 (61)	257 (49)	0.003
50–64 yrs	86 (39)	268 (51)	
Sex			
Female	99 (45)	124 (24)	<0.001
Male	121 (55)	401 (76)	
Alcohol abuse	39 (18)	130 (25)	0.030
Current smoking	123 (56)	304 (58)	0.530
Prior antibiotic therapy	18 (8)	182 (35)	<0.001
Clinical syndromes			
Pneumonia with empyema	33 (15)	36 (7)	<0.001
Pneumonia without empyema	149 (68)	331 (63)	
Others†	38 (17)	158 (30)	
Shock at presentation	15 (7)	107 (20)	<0.001
30-day mortality	11 (5)	107 (20)	<0.001
Antibiotic Resistance‡			
Penicillin			
Non-meningeal (MIC≥4 µg/mL)	4 (2)	12 (2)	0.688
Meningeal (MIC≥0.12 µg/mL)	36 (16)	180 (34)	<0.001
Cefotaxime/Ceftriaxone			
Non-meningeal (MIC≥2 µg/mL)	4 (2)	10 (2)	0.937
Meningeal (MIC≥1 µg/mL)	17 (8)	81 (15)	0.011
Erythromycin (MIC≥0.5 µg/mL)	33 (15)	124 (24)	0.009

Unless otherwise indicated, data are reported as number (percentage). MIC denotes minimum inhibitory concentration.

* Co-morbidities (some patients had more than one): HIV infection (n = 159), malignant disease (n = 143), chronic liver disease (n = 131), immunosuppressive therapy (n = 128), diabetes (n = 92), chronic pulmonary disease (n = 82), cardiovascular diseases (n = 45), transplant recipients (n = 29), chronic rheumatic disease or vasculitis (n = 20), asplenia (n = 19), end stage renal failure (n = 17), cerebrovascular diseases/degenerative brain diseases (n = 7).

† Other clinical syndromes [healthy (n) vs. co-morbidities (n)]: primary bacteraemia (3 vs. 42), meningitis (27 vs. 37), spontaneous bacterial peritonitis (0 vs. 50), abdominal/biliary tract (2 vs. 11), soft tissue infections (0 vs. 7); endocarditis (3 vs. 0); osteoarticular (2 vs. 5); otitis/sinusitis (1 vs. 6).

‡ According to the new Clinical Laboratory Standard Institute breakpoints. Resistance to other antibiotics (healthy vs. co-morbidities): tetracycline (12% vs. 26%); co-trimoxazole (20% vs. 41%) and ciprofloxacin (0.5% vs. 1%).

The incidence rates of IPD among adults (aged 18–64 years) in three periods (1996–2000 vs. 2001–2005 vs. 2006–2010) and according to PCV7 serotypes/non-PCV7 serotypes were: 3.5/5.0 vs. 2.9/6.8 vs. 2.5/9.2 episodes/100.000 (P<0.001) respectively.

Also, over the study period the proportion of IPD episodes in healthy adults increased: 18% (35/193) vs. 32% (78/245) vs. 35% (107/307), P<0.001.

There was an increase in non-PCV7 serotypes (periods: 1996–2000 vs. 2001–2005 vs. 2006–2010) among healthy adults: 66% (23/35) vs. 82% (64/78) vs. 86% (89/103), P = 0.02; and among patients with comorbidities: 57% (89/156) vs. 64% (106/165) vs. 75% (144/192), P = 0.002.

### Characteristics of healthy adults

As shown in Table 1, compared to patients with co-morbidities, the healthy adults had: younger age and higher proportion of women; more frequently pneumonia with empyema; and lower proportions of alcohol abusers, prior antibiotics, shock and mortality. The healthy adults also had lower antibiotic resistance rates.

**Figure 1:**
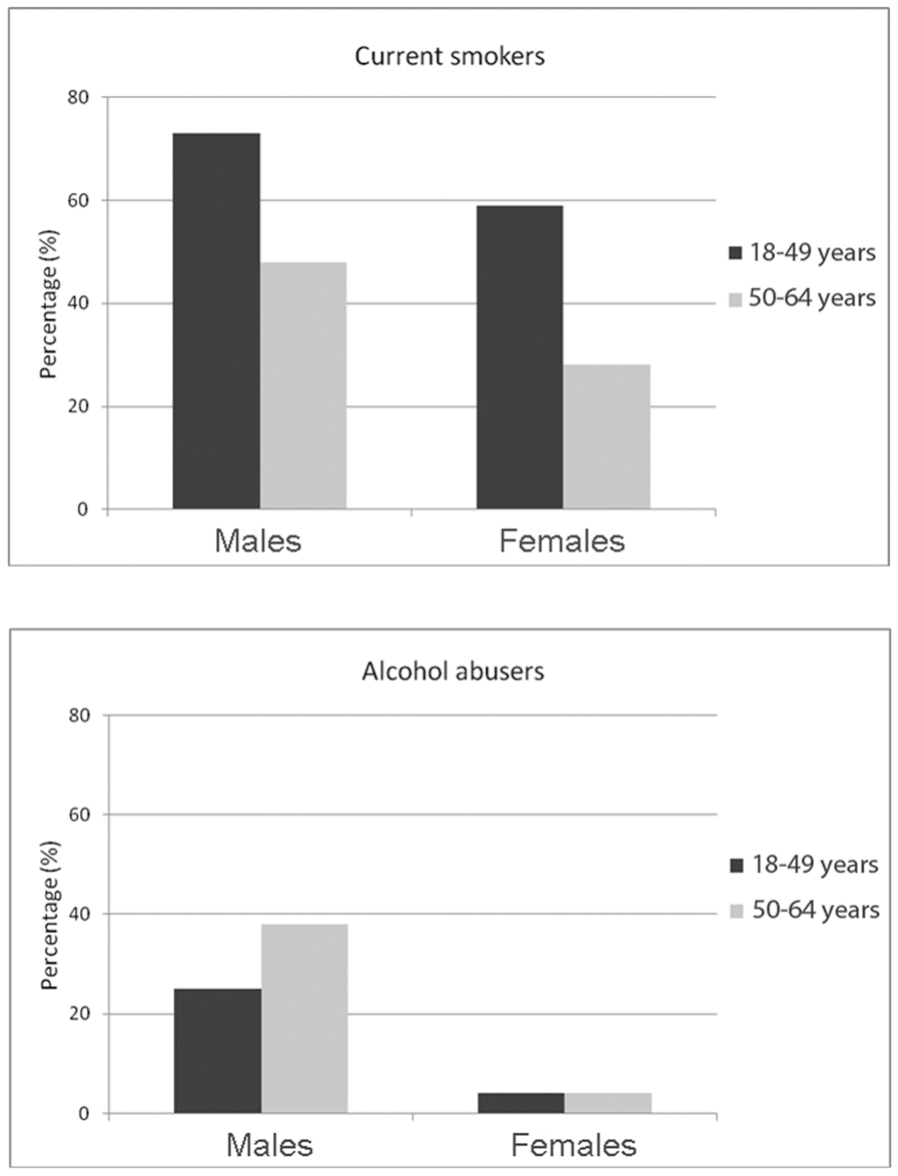
Percentage of current smokers and alcohol abusers among 220 healthy adults with invasive pneumococcal disease.

Of note, the healthy adults with IPD were often smokers (56%) or alcohol abusers (18%), and when stratified by gender and age group (18–49 and 50–64 years) these percentages were: smokers (men, 73% and 48%; women, 59% and 28%); and alcohol abusers (men, 25% and 38%; women, 4% and 4%), Figure 1.

In the subgroup of 549 patients with pneumonia, healthy adults (compared to patients with co-morbidities) had: higher proportion of empyema 18% (33/182) vs.10% (36/367), P = 0.006; and lower proportions of multilobar pneumonia 23% (42/182) vs. 34% (123/367), P = 0.011, and respiratory failure 34% (61/182) vs. 52% (192/367), P<0.001.

**Figure 2:**
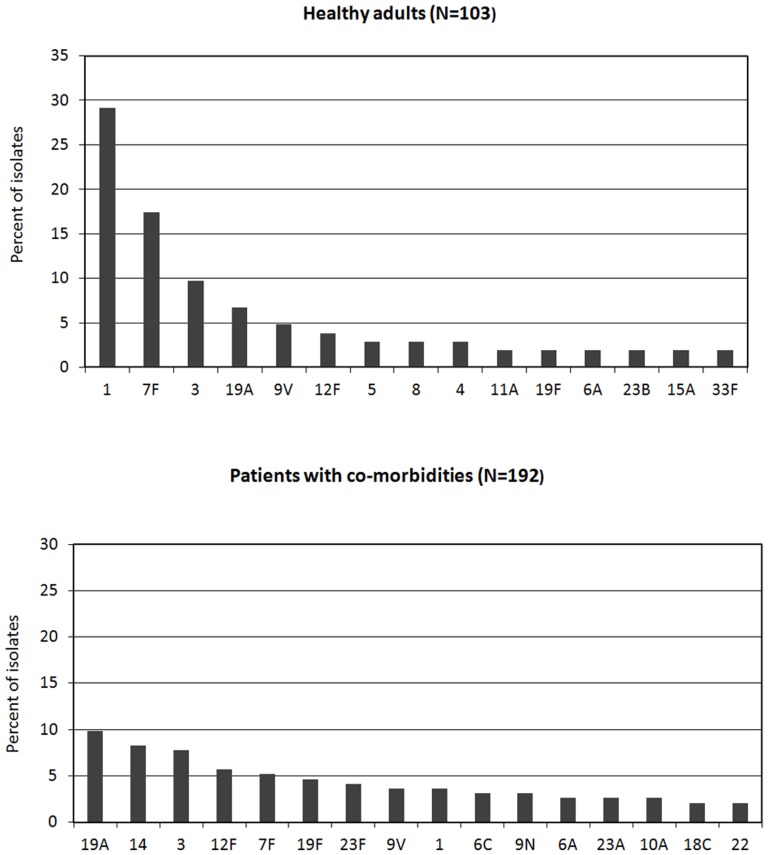
Serotype distribution among 295 pneumococcal isolates causing adult IPD in more recent years (period 2006–2010).

Healthy adults (compared to patients with co-morbidities) had higher proportions of serotypes 1,7F and 5, and lower proportions of serotype 19F, 23F and others (Table 2). The distribution of serotypes in more recent years (period 2006–2010) is shown in Figure 2. The vaccine serotype coverage (2006–2010) was higher in healthy adults than in patients with co-morbidities for PCV13 (82% vs. 56%, P<0.001) and for PCV10 (63% vs. 35%, P<0.001), while it was lower for PCV7 (14% vs. 25%, P = 0.022).

**Table 2:** Serotype distribution among 745 invasive pneumococcal isolates from adults (healthy vs. co-morbidities).

Serogroups/serotypes	Healthy N = 216*	Co-morbidities N = 513*	*P* value
	n° strains (%)		
1	55 (25)	24 (5)	<0.001
3	21 (10)	33 (6)	0.121
4	11 (5)	25 (5)	0.901
5	15 (7)	10 (2)	0.001
6A	5 (2)	12 (2)	0.984
6B	2 (1)	11 (2)	0.364
6C	1 (0.5)	8 (2)	0.458
7F	27 (13)	22 (4)	<0.001
8	7 (3)	20 (4)	0.668
9N	0	11 (2)	-
9V	7 (3)	30 (6)	0.143
11A	4 (2)	14 (3)	0.607
12	7 (3)	17 (3)	0.960
14	12 (6)	37 (7)	0.415
18C	3 (1)	14 (3)	0.420
19A	12 (6)	37 (7)	0.415
19F	3 (1)	30 (6)	0.006
23A	0	11 (2)	-
23F	2 (1)	27 (5)	0.006
Others†	22 (10)	120 (23)	<0.001

* A total of 745 IPD episodes. Out of 220 IPD in healthy adults, 216 (98%) were serotyped. Out of 525 IPD in patients with co-morbidities, 513 (98%) were serotyped.

† Others [healthy (n) vs. co-morbidities (n)]: 10A (2 vs. 10), 13 (0 vs. 3), 15A (3 vs. 9), 15B (0 vs. 9), 15C (0 vs. 3), 15F (0 vs. 4), 16F (0 vs 5), 17 (0 vs. 5), 18A (0 vs. 1), 18B (0 vs. 1) 20 (0 vs. 10), 21 (0 vs. 1), 22F (5 vs. 8), 23B (2 vs. 2), 24F (2 vs. 8), 25 (2 vs. 1), 28 (0 vs. 2), 29 (0 vs. 2), 31 (0 vs. 5), 32 (0 vs. 1), 33 (3 vs. 4), 34 (0 vs. 7), 35 (1 vs. 13), 37 (0 vs. 1), 38 (0 vs. 2), 48 (0 vs. 1), non-typable (2 vs. 2).

Of 79 serotype 1 isolates, 72 were genotyped, which were included into two genotypes related to ST304 (N = 15, Sweden^1^-ST304) and ST306 (N = 57, Sweden^1^-ST306). There was an emergence of ST306 during the study period: 43% (6/18 isolates) in 1996–2000, 91% (21/23) in 2001–2005 and 86% (30/35) in 2006–2010 (P = 0.001). Of 49 serotype 7F isolates, 48 were genotyped and shared a common genotype related to ST191 (Netherlands^7F^-ST191 clone). Of 25 serotype 5 isolates, 22 were genotyped and shared a common PFGE type related to Columbia^5^-ST289 clone and associated with two STs (ST289 and ST1223).

### Multivariate analyses

Tables 1 and 2 show the clinical characteristics of IPD and serotypes. In a multiple logistic regression model, those variables independently associated with healthy adults (compared to patients with co-morbidities) were: younger age (≤49 yrs) [OR = 1.85, 95%CI 1.29–2.65, P = 0.001], female sex [OR = 2.56, 95%CI 1.76–3.72, P<0.001], presentation with pneumonia plus empyema [OR = 2.14, 95%CI 1.19–3.85, P = 0.010], serotype 1 [OR = 6.86, 95%CI 3.99–11.81, P<0.001], serotype 5 [OR = 5.53, 95%CI 2.33–13.11, P<0.001], and serotype 7F [OR = 3.82, 95%CI 2.04–7.13, P<0.001].

### Mortality

As expected, the healthy adults with IPD had a lower mortality rate than patients with co-morbidities (Table 1), and this persisted significantly lower after adjustment for age, gender, clinical presentation and serotypes (adjusted OR = 0.31, 95%CI 0.16–0.62, P = 0.001).

Of note, no deaths occurred among the 79 patients with serotype 1 IPD. However, 12% (6/49) of patients with serotype 7F and 8% (2/25) of patients with serotype 5, died.

Table 3 shows the 11 healthy adults with IPD who died. Early death occurred in 4 patients (2 with fulminant sepsis and 2 with meningitis). Two patients with endocarditis, without underlying cardiac disease, had a history of alcohol abuse and one of them died because of complications of cardiac surgery and the other due to a nosocomial infection. A 19-year old boy who presented with progressive bilateral pneumonia caused by serotype 3 died. The remaining 4 patients had extensive bacteremic pneumonia caused by serotypes 14 or 7F. In all cases the pneumococcal isolates were susceptible to the antibiotics administered.

**Table 3:** Summary of the 11 healthy adults with invasive pneumococcal disease who died.

Patient	Sex/Age (yr)	Alcoholabuse	Current smoking	Clinical syndrome	Source of isolate	Shock at presentation	Antibiotic therapy	Days to death	Serotype
1	M/52	Yes	Yes	Endocarditis/Meningitis	blood	No	CTX	29	10A
2	M/42	Yes	Yes	Endocarditis	blood	Yes	CRO	16	4
3	M/19	No	Yes	Pneumonia	blood	Yes	CRO	9	3
4	F/53	No	No	Pneumonia	blood	No	CRO+LEVO	30	14
5	F/58	No	No	Pneumonia	blood	Yes	CRO	29	7F
6	M/49	Yes	No	Pneumonia	blood	No	LEVO	10	14
7	M/36	No	No	Sepsis	blood	Yes	CRO	1	6A
8	F/40	No	Yes	Pneumonia	blood	Yes	CRO	12	7F
9	M/37	No	No	Meningitis	Blood/CSF	No	VAN+RIFA	4	3
10	M/39	No	Yes	Sepsis	blood	Yes	CTX	1	ND
11	M/59	No	Yes	Meningitis	Blood/CSF	Yes	CTX	3	3

Abbreviations: M, male; F, female; CTX, cefotaxime; CRO, ceftriaxone; LEVO, levofloxacin; VAN, vancomycin; RIFA, rifampin; ND, not done.

### Pneumonia and empyema

Among 549 patients with pneumonia, 69 (13%) had associated empyema with isolation of *S.pneumoniae* in pleural fluid (“empyema group”), and 480 (87%) had pneumonia without empyema (“pneumonia group”) (Table 4).

**Table 4:** Pneumococcal pneumonia in 549 adults: A comparison between patients with pneumonia and empyema (Empyema group) and those with pneumonia without empyema (Pneumonia group).

	Empyema group N = 69	Pneumonia group N = 480	*P*
Age, mean+/−SD, years	46.83 (+/−12.83)	46.74 (+/−12.23)	0.959
18–49 yrs	33 (48)	261 (54)	0.308
50–64 yrs	36 (52)	219 (46)	
Sex			
Female	21 (30)	146 (30)	0.998
Male	48 (70)	334 (70)	
Underlying diseases			
Healthy	33 (48)	149 (31)	0.006
Co-morbidities*	36 (52)	331 (69)	
Alcohol abuse	13 (19)	115 (24)	0.347
Current smoking	40 (58)	301 (63)	0.448
Multilobar pneumonia	20 (29)	145 (30)	0.968
Respiratory failure	33 (48)	220 (46)	0.827
Shock at presentation	7 (10)	88 (18)	0.060
30-day mortality	6 (9)	71 (15)	0.198
Serotypes†			
Serotype 1	17 (25)	56 (12)	0.003
Serotype 3	8 (12)	35 (7)	0.162
Serotype 7F	4 (6)	41 (9)	0.320
Others	39 (57)	340 (72)	0.013

Unless otherwise indicated, data are reported as number (percentage).

* Co-morbidities in the empyema group (n) vs. pneumonia group (n) (some patients had more than one): HIV infection (9 vs. 116), malignant disease (15 vs. 85), chronic liver disease (4 vs. 55), immunosuppressive therapy (11 vs. 78), diabetes (5 vs. 57), chronic pulmonary disease (7 vs. 62), cardiovascular disease (2 vs. 33), chronic rheumatic disease or vasculitis (0 vs. 15), asplenia (1 vs. 3), end stage renal failure (3 vs. 12), transplant recipients (1 vs. 17), cerebrovascular or degenerative brain disease (1 vs. 5).

† The most common serotypes in patients of empyema group (68/69 serotyped) were: serotype 1 (25%), 3 (12%), 7F (6%), 5 (4%), 19A (4%), 19F (4%), 23F (4%), 4 (3%), 6A (3%), 6B (3%), 8 (3%), 9V (3%), 18C (3%), 23A (3%) and 22 (3%).

The most common serotypes in patients of pneumonia group (472/480 serotyped) were: serotype 1 (12%), 7F (9%), 14 (8%), 19A (8%), 3 (7%), 4 (6%), 9V (5%), 5 (5%), 8 (5%), 12F (4%), 23F (3%), 11A (3%), 18C (3%) and 19F (2%).

Empyema occurred more frequently in healthy adults and was often associated with serotype 1, particularly the clonal-type Sweden^1^-ST306 (15 of 17 cases).

## Discussion

In our study, about one third of adults (aged 18–64 years) with IPD were healthy, and we observed an increase in IPD caused by non-PCV7 serotypes, particularly the serotypes 1, 7F and 5. Of special concern has been the spread of some specific clonal-types, such as Sweden^1^-ST306 of serotype 1 that was strongly associated with empyema.

Our findings are consistent with some reports considering serotypes 1, 5 and 7F as primary pathogens with a high invasive disease potential, because they often cause IPD in children but are associated with a brief duration of colonization and are rarely found in the nasopharynx. [22]–[25]. The serotype 1 clone (Sweden^1^-ST306) seen in our study was first identified in Sweden in 1990's with a subsequent spread to other European countries [24], [26]–[31].

Smokers and alcohol abusers are at greater risk for IPD [5], [8]. Indeed, our healthy adults with IPD were frequently smokers or alcohol abusers; and we decided not to exclude them from the healthy adults group in order to identify factors suitable for future vaccination strategies. Thus, our results suggest that avoiding smoking and alcohol abuse and reinforcing the pneumococcal vaccination (CDC recommendations) for active smokers and alcohol abusers [9] may reduce the IPD rates in adults. These data support the initiative of Joint Commission's tobacco-cessation interventions in patients attending health care visits [32].

The increased proportion of women among our healthy adults with IPD may reflect that healthy women have often contact with children who may facilitate the transmission of the pneumococcus and that they often had a history of smoking which may predispose to IPD.

Pneumococcal strains often harbour antibiotic resistance [15], [33], [34]. Our healthy adults had lower antibiotic resistance rates than patients with co-morbidities (Table 1) since they were often infected with susceptible serotypes and received prior antibiotics less frequently. Most non-meningeal pneumococcal isolates were penicillin-, and ceftriaxone-susceptible, which support the use of these classic beta-lactams in the treatment of pneumococcal infections [15], [33].

Occasionally, we have seen healthy adults with IPD who died despite appropriate antibiotic therapy. It should be noted that few of them were alcohol abusers but several others denied alcohol abuse and presented with severe clinical syndromes (Table 3). It is possible that unknown underlying host factors or increased virulence of some serotypes or clonal-types may have played a role in the patient's death.

An important finding of our study is the high prevalence of serotype 1 in healthy adults, particularly the clonal-type Sweden^1^-ST306, which was strongly associated with empyema, as occurred in older children [27], [28]. The intrinsic characteristics of serotype 1 capsular polysaccharide structure, surface proteins properties or the pneumolysin might explain some differences in the clinical presentation [25], [35].

Also, it is unknown why serotype 1 is more prevalent in older children and healthy adults than in patients with co-morbidities. We can speculate that patients with co-morbidities, who often undergo antibiotic pressure and may have local and systemic immunodeficiencies, are more predisposed to be colonized/infected with multi-resistant serotypes -with low invasive disease potential- such as 19F and 23F, and because of a phenomenon of “competition among serotypes in the nasopharyngeal niche” they could be less predisposed to acquire other serotypes such as serotype 1. On the contrary, healthy adults who are not usually nasopharyngeal carriers could be more predisposed to be infected with invasive serotypes, which act as primary pathogens, such as serotype 1.

Some reports have shown a low mortality of serotype 1 IPD [36], [37]. Interestingly, none of our 79 patients with IPD caused by serotype 1 died, which may be related to host's characteristic (e.g., healthy people) or differences in pneumococcal-dependent factors (e.g., capacity to generate a host inflammatory response) [25].

It is well known the widely geographical variation of serotype 1, which is quite prevalent in several European countries but less common in USA and Canada [25]. Also, several epidemics of serotype 1 IPD have been reported in South American, Asian and African countries [25], [38]. It is possible that genetic and environmental factors as well as clonal-type dissemination may explain some of these differences. For example, cases of serotype 1 IPD reported in USA were often caused by a different clonal-type, the ST227 [39].

We also found a high prevalence of serotypes 7F and 5 among healthy adults. The characteristics of IPD caused by these serotypes were quite similar to those of serotype 1, except that were less strongly associated with empyema and some of them died. Serotype 3 was also prevalent in healthy adults as well as in patients with co-morbidities (Table 2).

We found that the serotype coverage of the recently introduced PCV13 was higher in healthy adults than in patients with co-morbidities, which reflects a different serotype distribution. We do not know if in areas with increased prevalence of serotype 1 (which affects more frequently older children and healthy adults), the use of PCV13 for children <2 yrs will be associated with a decline of IPD in healthy adults (by herd protection). Recent studies have evaluated the cost-effectiveness of PCV13 compared to PPV23 in adult vaccination, and suggest an advantage of the PCV13 with the assumption that it would be effective against non-bacteremic pneumococcal pneumonia [40].

Although our study refers to a large cohort of patients with IPD, it may have some limitations: 1) the study was performed in Barcelona and these results may not be entirely extrapolated to other geographical areas; 2) our study did not show specific incidence rates of IPD in healthy people and in patients with co-morbidities since we do not know these denominators; and 3) we could not investigate other possible risk factors for IPD such as viral co-infections, nor the close contact with young children.

In conclusion, our study shows that IPD often occurs in healthy adults, particularly smokers and alcohol abusers, and frequently presents with pneumonia and empyema associated with virulent clones of non-PCV7 serotypes. Thus, implementing tobacco and alcohol abuse-cessation measures and a proper pneumococcal vaccination, which may include a broader spectrum conjugate vaccines such as PCV13, in active smoker and alcohol abusers may diminish the burden of IPD in adults.
